# Metabolic pathway activation and immune microenvironment features in non-small cell lung cancer: insights from single-cell transcriptomics

**DOI:** 10.3389/fimmu.2025.1546764

**Published:** 2025-02-28

**Authors:** Yanru Liu, Hanmin Liu, Ying Xiong

**Affiliations:** ^1^ Department of Pediatric Pulmonology and Immunology, West China Second University Hospital, Sichuan University, Chengdu, China; ^2^ Key Laboratory of Birth Defects and Related Diseases of Women and Children (Sichuan University), Ministry of Education, Chengdu, China; ^3^ Department of Pediatric Chengdu Women’s and Children’s Central Hospital, School of Medicine, University of Electronic Science and Technology of China, Chengdu, China

**Keywords:** metabolic pathway, non-small cell lung cancer, weighted gene co-expression network analysis, risk signature, tumor microenvironment

## Abstract

**Introduction:**

In this study, we aim to provide a deep understanding of the tumor microenvironment (TME) and its metabolic characteristics in non-small cell lung cancer (NSCLC) through single-cell RNA sequencing (scRNAseq) data obtained from public databases. Given that lung cancer is a leading cause of cancer-related deaths globally and NSCLC accounts for the majority of lung cancer cases, understanding the relationship between TME and metabolic pathways in NSCLC is crucial for developing new treatment strategies.

**Methods:**

Finally, machine learning algorithms were employed to construct a risk signature with strong predictive power across multiple independent cohorts. After quality control, 29,053 cells were retained, and PCA along with UMAP techniques were used to distinguish 13 primary cell subpopulations. Four highly activated metabolic pathways were identified within malignant cell subpopulations, which were further divided into seven distinct subgroups showing significant differences in differentiation potential and metabolic activity. WGCNA was utilized to identify gene modules and hub genes closely associated with these four metabolic pathways.

**Results:**

Our analysis showed that DEGs between tumor and normal tissues were predominantly enriched in immune response and cell adhesion pathways. The comprehensive examination of our model revealed substantial variations in clinical and pathological characteristics, enriched pathways, cancer hallmarks, and immune infiltration scores between high-risk and low-risk groups. Wet lab experiments validated the role of KRT6B in NSCLC, demonstrating that KRT6B expression is elevated and it stimulates the proliferation of cancer cells.

**Discussion:**

These observations not only enhance our understanding of metabolic reprogramming and its biological functions in NSCLC but also provide new perspectives for early detection, prognostic evaluation, and targeted therapy. However, future research should further explore the specific mechanisms of these metabolic pathways and their application potentials in clinical practice.

## 1 Introduction

On a global scale, the leading cause of deaths related to cancer is lung cancer (1). From a histological perspective, the two main types of lung cancer are categorized as non-small cell lung cancer (NSCLC) and small cell lung cancer. According to epidemiological data, NSCLC accounts for roughly 85% of all lung cancer diagnoses and can be further subdivided into three major subtypes: adenocarcinoma, large cell lung cancer, and squamous cell carcinoma (2–4). NSCLC’s development is influenced by multiple factors, encompassing a sophisticated interaction between environmental and personal variables, including smoking, secondhand smoke, occupational exposure, air pollution, genetic susceptibility, chronic lung diseases, age, gender, dietary habits, and radiation exposure. Among these, active or passive exposure to tobacco smoke is indisputably a key factor in the development of NSCLC (5). In terms of pathological characteristics, among the subtypes of NSCLC, adenocarcinoma is the most frequently observed, typically originating from alveolar cells in the epithelium of smaller airways and often presenting in the peripheral lung; squamous cell carcinoma is primarily found in the central bronchial regions; large cell carcinoma is a highly malignant tumor, usually diagnosed at later stages (3, 6). Even with substantial advancements in early detection and treatment methods over past few decades, the percentage of NSCLC patients who survive for five years remains below 20% (7). Currently, with the ongoing development of biomarkers, targeted therapies are increasingly being utilized in NSCLC patients (8). However, data reveal that fewer than 25% of patients benefit from targeted therapies, and the development of resistance during treatment is nearly ubiquitous (9). Therefore, it is pivotal to delve deeper into the molecular mechanisms behind NSCLC and to identify novel therapeutic targets.

Research has extensively shown that cellular biological functions rely heavily on glycolysis, lipid metabolism, and nucleotide metabolism. In recent years, the significance of metabolic reprogramming in cancer progression has garnered growing attention. Glycolysis/Gluconeogenesis is a central metabolic pathway in cellular energy metabolism. Glycolysis is the process by which cells generate energy by breaking down glucose into pyruvate, primarily occurring in the cytoplasm and accompanied by the production of small amounts of ATP. Gluconeogenesis, the reverse of glycolysis, primarily occurs in the liver and kidneys (10). Malignant cells often exhibit the “Warburg effect,” where they preferentially rely on glycolysis for energy production even under aerobic conditions. This phenomenon not only supports rapid tumor cell proliferation but also leads to lactate accumulation, which acidifies the tumor microenvironment (TME), thereby promoting metastasis and the development of resistance (11, 12). Glycosaminoglycan (GAG) degradation refers to the enzymatic breakdown of GAGs into smaller fragments. GAGs are complex polysaccharides found in the extracellular matrix, as well as the surfaces of cell, playing roles in cell adhesion, signaling, and other functions. In the TME, the structure and function of the extracellular matrix can be modified by the degradation products of GAGs, which in turn affects tumor metastasis and invasion (13). Glycosphingolipids are important cell membrane lipids involved in signaling, cell recognition, and various other biological processes. The synthesis of glycosphingolipids in the ganglio series is essential for both the nervous system and the TME. Research has demonstrated that changes in glycosphingolipids can affect tumor cell growth and metastasis, particularly in NSCLC, where abnormal expression of gangliosides has been associated with increased tumor cell invasiveness and drug resistance (14). The biosynthesis of the globo and isoglobo series of glycosphingolipids involves a distinct set of enzymatic reactions. These molecules, although structurally different from gangliosides, also participate in various cellular functions. In tumor cells, changes in glycosphingolipid expression may affect intercellular interactions, cell adhesion, and migration (15). We can reasonably infer that these metabolic pathways are not only critical for cellular energy metabolism and signaling but also closely linked to the beginning and advancement of NSCLC. In-depth study of the regulatory mechanisms and activation levels of these pathways in NSCLC could offer novel perspectives for early detection, prognosis evaluation, and targeted treatments.

Analysis of single-cell RNA sequencing (scRNA-seq) was conducted by us, using multiple public datasets to explore the heterogeneity of gene expression and metabolic features across different cell subpopulations in NSCLC. Within the malignant cell subpopulations, we identified four highly activated metabolic pathways. Through the application of Weighted Gene Co-expression Network Analysis (WGCNA), we discovered co-expression modules and pivotal hub genes that were notably correlated with the four metabolic pathways. Leveraging data sourced from NSCLC patients, we were able to establish a risk signature that exhibited strong predictive capabilities across diverse independent cohorts. Ultimately, we embarked on a comprehensive analysis of the model, encompassing clinical-pathological characteristics, enrichment analysis, cancer hallmark pathways, and immune infiltration scores, thereby revealing substantial disparities between high-risk and low-risk groups across multiple facets. These observations not only deepen our understanding of metabolic reprogramming and its biological functions in NSCLC but also offer new perspectives for early detection, prognostic evaluation, and targeted therapy.

## 2 Material and methods

### 2.1 Dataset acquisition and preprocessing

We first retrieved the bulk RNA sequencing (bulk RNA-seq) datasets TCGA-LUAD and TCGA-LUSC for NSCLC from Cancer Genome Atlas (TCGA, https://portal.gdc.cancer.gov/) via “TCGAbiolinks” R package. For the aim of facilitating better gene differential expression analysis between samples, the transcriptomic data was transformed into Transcripts Per Million (TPM) format. Additionally, we obtained the bulk RNA-seq datasets GSE31210 and GSE37745 from Gene Expression Omnibus (GEO, https://www.ncbi.nlm.nih.gov/geo/) database using “GEOquery” R package. Finally, we collected three independent scRNA-seq datasets, GSE143423, GSE117570, and GSE150660, from Tumor Immune Single-cell Hub 2 (TISCH2, http://tisch2.ca) database. The datasets utilized in our study are publicly accessible and allow unrestricted use without requiring additional ethical approval. Our data acquisition and analysis processes were in compliance with relevant regulations. All open source public databases used in this study are available for unrestricted access and use and do not require additional ethical approval. Our data acquisition and analysis processes follow the relevant regulations.

### 2.2 Single-cell sequencing data analysis

We use the “harmony” R package for multi-copy consolidation and batch processing. Our quality control standard is: nFeature_RNA < 9000 & percent.mt < 25. The gene and transcript counts of the three independent datasets (GSE143423, GSE117570, and GSE150660) were visualized via violin plots. Variability in gene expression across all cells was further explored through a variance plot. Next, dimensionality reduction was achieved by utilizing Uniform Manifold Approximation and Projection (UMAP) in our study, identifying distinct cell subpopulations within the datasets and visualizing their distribution on UMAP plots. Following this, Principal Component Analysis (PCA) was conducted to cluster data, with a resolution of 0.6, and the resulting principal components (PCs) were visualized using bubble plots. Based on these PCs, we displayed the cell distribution in UMAP space for each dataset (GSE143423, GSE117570, and GSE150660).

We employed the SingleR method for automated cell-type annotation, identifying several major cell types. For each cell type, we obtained marker genes and analyzed their expression levels, visualizing the results accordingly. Through using the “scMetabolism”R package, we performed a metabolic investigation of the cell subpopulations. The activity levels of crucial metabolic pathways associated with each major cell type were visualized in bubble plots and subjected to comparative analysis. Four metabolic pathways, which were upregulated in malignant cells, were selected for further analysis. The differences in pathway activity across major cell types were illustrated using box plots. Next, we conducted unsupervised clustering through “ConsensusClusterPlus” R package and created a Consensus Matrix heatmap. The Proportion of Ambiguous Clustering (PAC) plot was used to determine the optimal number of clusters (K), identifying the K value corresponding to the lowest point on the y-axis. Next, we used scMetabolism to analyze the four metabolic pathways in each cluster. The “Seurat 4.4” R package was utilized to identify genes with elevated expression in cluster C2 using its FindMarkers function. Score using AddModuleScore() built into the Seurat R package. Subsequently, these genes underwent Gene Set Enrichment Analysis (GSEA) and Over Representation Analysis (ORA) through the “clusterProfiler” package. The pathways analyzed encompassed Gene Ontology (GO) terms and Kyoto Encyclopedia of Genes and Genomes (KEGG) pathways. The bar plots clearly depicted the upregulation and downregulation of these pathways within cluster C2.

We further analyzed the malignant cell subpopulation, first performing UMAP dimensionality reduction to identify several subclusters. Differentially expressed genes in each subcluster were highlighted. The CytoTRACE2 method was used to predict the differentiation potential of each subcluster. Furthermore, by employing monocle2, we performed pseudotime analysis, examining the distribution of subclusters along the cell trajectory to infer the evolutionary order and origin of the subpopulations. The relative activity of the four upregulated metabolic pathways in the malignant cell subclusters was tracked over pseudotime.

### 2.3 Analyzing differential expression and assessing enrichment

To identify differentially expressed genes (DEGs) between cancerous and normal tissues, on the combined datasets of TCGA-LUAD and TCGA-LUSC, we applied the R package “limma” with the criteria of log_2_|Fold change| (log_2_FC) greater than 1 and an adjusted p-value below 0.05. The outcomes were then graphically represented through volcano plots and heatmaps. We then performed KEGG pathway enrichment analysis for the DEGs, presenting the top 20 statistically significant pathways in bar plots. Otherwise, we performed GO analysis on the DEGs and presented pathways which are belonging to the five most significant enrichments within the categories of Biological Process (BP), Cellular Component (CC), and Molecular Function (MF).

### 2.4 WGCNA

In the combined TCGA-LUAD and TCGA-LUSC datasets, Gene Set Variation Analysis (GSVA) was initially employed to evaluate four metabolic pathways that displayed notable activation, with the findings illustrated in a heatmap. Following this, we chose an optimal soft threshold β to construct a scale-free network based on the criteria of scale independence and mean connectivity. To present the results of hierarchical clustering, a dendrogram was utilized, showcasing several co-expression modules, each assigned a unique color. We then investigated the relationship between these modules and the four metabolic pathways, illustrating the results through another heatmap. We set the requirements for Module Membership (MM) to be greater than 0.4 and Gene Significance (GS) over 0.3. Our main attention was directed towards the modules showing significant negative correlations with the pathways. For every identified module, we examined the relationship between gene significance and module membership to identify crucial hub genes. In the concluding phase, we conducted GO enrichment analysis on the hub genes recognized within the modules, presenting the outcomes with a lollipop plot.

### 2.5 Establishing a risk signature for NSCLC patients utilizing the combined TCGA cohort

We determined the common hub genes shared among those from WGCNA, marker genes obtained through single-cell analysis, and DEGs, and illustrated their overlap with a Venn diagram. Using the intersected genes, we determined Cox regression analysis to evaluate their prognostic significance. Next, we applied the analysis of Least Absolute Shrinkage and Selection Operator (LASSO) regression to these genes, utilizing the combined TCGA-LUAD and TCGA-LUSC datasets as the training cohort. The selected parameter is “cvfit$lambda.min”. The selection of optimal prognostic genes was based on the determination of the optimal λ parameter. Subsequently, the risk score for each patient was calculated using the following formula:


$$RiskScore=\sum_{i=1}^{n}Exp_{gene_{i}}*\beta_{i}$$

$Exp_{gene_{i}}$ represents expression levels of model genes, *β_i_* denotes the corresponding coefficients of these genes. We employed the GSE31210 and GSE37745 datasets as external validation cohorts. The datasets were stratified into high-risk and low-risk groups, with the division based on the median risk score. Kaplan-Meier (KM) curves were then generated to assess prognosis of these groups. Additionally, Receiver Operating Characteristic (ROC) curves were constructed to evaluate predictive accuracy of prognostic model at 1-year, 3-year, and 5-year time points.

### 2.6 Correlation analysis based on risk signature

We followed the same stratification approach to divide the cohorts into high-risk groups and low-risk groups. Initially, our investigation focused on examining the variations in expression levels of model genes, along with the clinical-pathological features, across the two defined risk groups. Subsequently, for high-risk group, we used the “limma” package to identify DEGs, followed by GSEA using the gseKEGG function from the “clusterProfiler” package. From the Molecular Signatures Database (MSigDB) available at https://www.gsea-msigdb.org/gsea/msigdb/, we obtained cancer hallmark gene sets and applied GSVA to quantify the disparities in cancer hallmark scores among the risk groups. Utilizing the CIBERSORT algorithm, facilitated by the “IOBR” R package, we evaluated the infiltration levels of immune cells in both groups. Furthermore, we conducted a comparative analysis of the expression profiles of T cell exhaustion markers and M2 polarization regulators across the risk groups, while also employing the Tumor Immune Dysfunction and Exclusion (TIDE) score to forecast the effectiveness of immune checkpoint inhibitors. Additionally, we investigated the differential expression of pro-tumor immune cells, specifically Cancer-Associated Fibroblasts (CAFs) and Myeloid-Derived Suppressor Cells (MDSCs), between the two groups. Lastly, we performed a drug sensitivity analysis on four drugs (Bortezomib, Olaparib, Tamoxifen, and Axitinib) using “OncoPredict” R package.

### 2.7 Cell culture and transfection

In this experiment, we utilized the immortalized human normal lung epithelial cell line BEAS-2B, along with the lung cancer cell lines A549, NCI-H1299, NCI-H1975, and NCI-H358, all of which were obtained from the Cell Bank of the Chinese Academy of Sciences. The cell lines were cultured in Roswell Park Memorial Institute 1640 (RPMI-1640, HyClone, USA) medium, supplemented with 10% fetal bovine serum (FBS, BI, Israel), 100 U/ml penicillin (HyClone, USA), and 100 µg/ml streptomycin (HyClone, USA). To ensure they stayed in the logarithmic phase of growth, the cells were kept in an incubator maintained at 37°C with 5% CO_2_ and high humidity. Cell growth was monitored daily to confirm optimal growth conditions, and the cells were subcultured every 24 hours.

Two of the cell lines underwent transfection experiments. siRNA sequences targeting KRT6B expression in A549 and NCI-H358 cells were designed by a commercial biotechnology company (Sangon, China), with si-negative control (NC) used as a reference. To initiate the transfection, A549 and NCI-H358 cells were trypsinized, suspended in complete growth medium at a density of 1×10^5^cells per well, and plated in 6-well plates, with each well supplemented with 2 ml of complete medium. After cells adhered to the surface, siRNA and the transfection reagent PolyFast (Catalog No. HY-K1014, MCE, USA) were mixed according to the manufacturer’s recommendations and incubated at room temperature for 15 minutes. The mixture was then evenly pipetted into the corresponding wells. The medium was replaced 6 hours post-transfection, and subsequent experiments were conducted 48 hours after transfection.

### 2.8 Total RNA extraction and RT-qPCR

We have investigated the research status of model genes in the field of lung cancer, and the results show that only KRT6B gene has not been studied experimentally. Therefore, this paper reveals for the first time the role of KRT6B gene in lung cancer. To examine the variations in mRNA expression levels of KRT6B, we utilized RT-qPCR across five cell lines and to verify the effect of KRT6B knockdown in A459 and NCI-H358 cell lines. Initially, cells were detached from six-well plates using trypsin (HyClone, USA) and subjected to multiple PBS washes followed by low-speed centrifugation, after which the supernatant was discarded. According to the manufacturer’s instructions, cells were lysed using an appropriate amount of Trizol reagent (Takara, Japan). After obtaining the cell lysate, it was kept on ice for a duration of 5 minutes, whereupon 200 μl of chloroform sourced from Tokyo Chemical Industry (Japan) was added successively, followed by an identical volume of isopropanol also from Alfa Aesar (Thermo Fisher Scientific, USA), and finally anhydrous ethanol, again supplied by Sigma-Aldrich (USA). Each reagent was thoroughly mixed before centrifugation at 4°C and incubation on ice for 15 minutes. Following the removal of all organic solvents, the RNA pellet was allowed to dry in the air for 20 minutes. Subsequently, the RNA was resuspended in 15 μl of DEPC-treated water, and its concentration was quantified using a Nanodrop 2000 spectrophotometer (Thermo, USA). Subsequently, utilizing the PrimeScript RT Reagent Kit from Thermo Fisher Scientific (USA), the genomic DNA was eliminated, subsequently enabling the production of cDNA through the process of reverse transcription. For the qPCR assay, Prior to the experiment, the cDNA samples were blended with SYBR GreenER Supermix (Bio-Rad Laboratories, USA) in accordance with the manufacturer’s directives. Quantitative real-time PCR amplification was executed utilizing the CFX96 Touch Real-Time PCR Detection System (Bio-Rad Laboratories, USA). The choice of all reaction conditions was informed by the recommendations stipulated in the SYBR GreenER Supermix (Bio-Rad Laboratories, USA) user manual. The relative abundance of KRT6B was determined by employing the 2^–ΔΔCt^ methodology, with β-actin serving as the normalizing denominator for expression levels.

### 2.9 CCK8 assay

To evaluate how the knockdown of KRT6B influences the proliferation rates of A549 and NCI-H358 cell lines, we utilized the CCK8 assay as our experimental approach. After transfection of the two cell lines for 48 hours, the cells underwent trypsinization using trypsin sourced from KeyGEN BioTECH (China), followed by their uniform distribution in a complete growth medium. Based on cell counting results, cells were then transferred to a 96-well plate (5,000 cells/well). Three replicates were set up for each group to ensure result accuracy. After the cells adhered to the surface, according to the manufacturer’s guidelines, CCK8 reagent (Dojindo Laboratories, Japan) was blended with complete culture medium at ambient temperature to attain a final volume of 200 μl per well. This mixture was promptly dispensed into each well, and the plate was securely wrapped in aluminum foil to shield it from light exposure. After a duration of 1.5 hours, the absorbance was quantified at a wavelength of 450 nm utilizing a microplate spectrophotometer. This process was repeated at 24, 48, 72, and 96-hour time points to monitor cell proliferation.

### 2.10 Statistical analysis

In our research, we employed the Kaplan-Meier (KM) methodology to perform survival analysis, and utilized the log-rank test to compare survival differences between high-risk and low-risk groups. Additionally, we computed the area under the curve (AUC) value, with an AUC greater than 0.55 considered indicative of good discriminative ability. We used the “survival” package to analyze the Cox proportional risk model. In addition, to better process and analyze gene expression data, we used the “survminer” package, which provides functions for visualization of survival analysis results and is able to simplify the presentation of results in Cox models, including hazard ratio (HR) and confidence interval (CI) extraction. A p value < 0.05 was considered statistically significant (* p < 0.05; ** p < 0.01; *** p < 0.001; **** p < 0.0001). The statistical analyses conducted in this study were all carried out utilizing the R programming environment, specifically version 4.3.1.

## 3 Results

### 3.1 Single-cell sequencing data analysis

**Figure 1:**
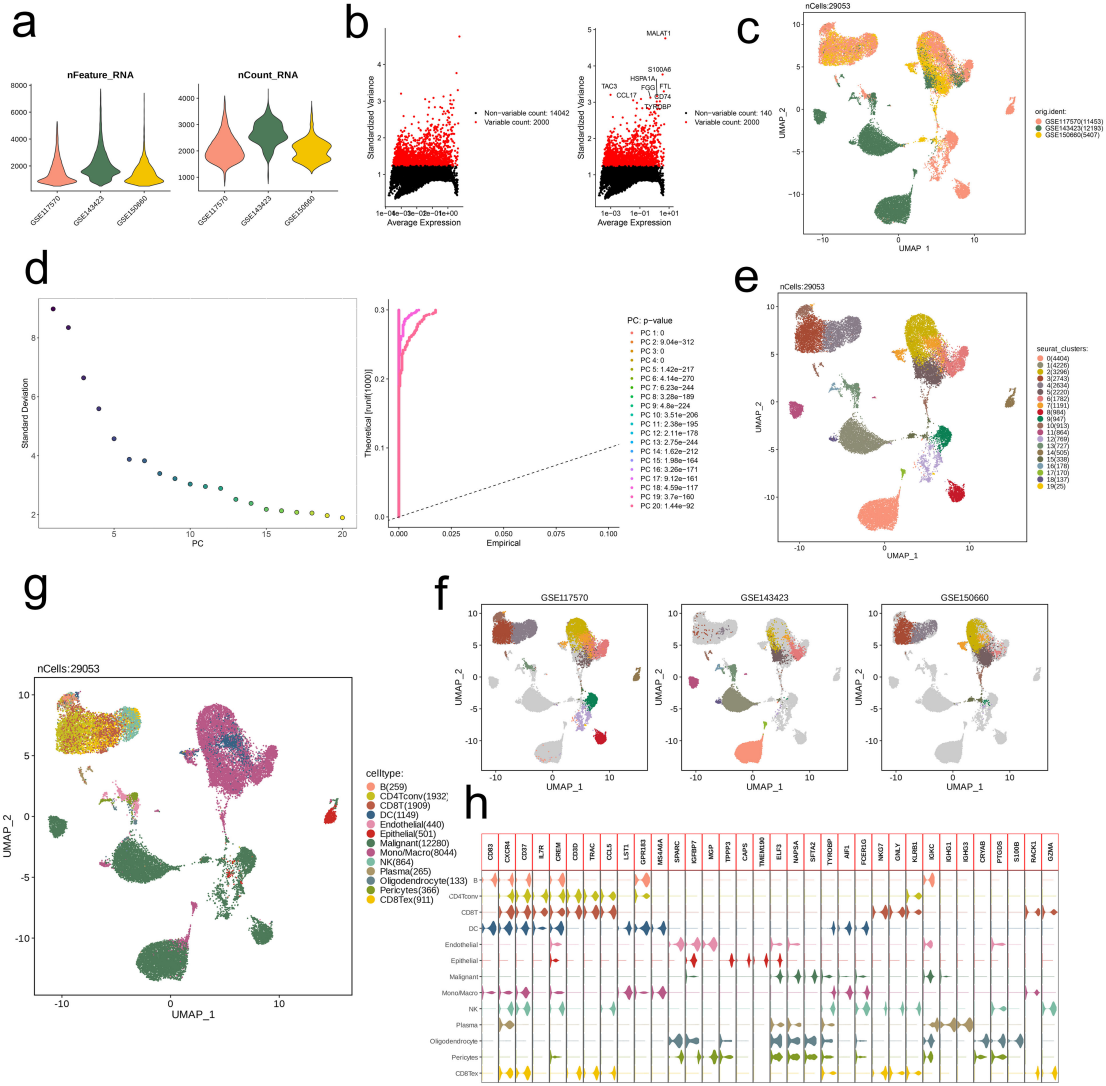
Identification of 13 cell clusters with diverse annotations revealing high cellular heterogeneity in NSCLC based on single-cell RNA-seq data. **(a)** After quality control of scRNA-seq, 29053 core cells were identified. **(b)** The variance diagram shows the variation of gene expression in all cells of NSCLC. The red dots represent highly variable genes and the black dots represent non-variable genes. **(c)** UMAP showed a clear separation of cells in NSCLC. **(d)** PCA identified the top 20 PCs at p < 0.05. **(e)** The UMAP algorithm was applied to the top 20 PCs for dimensionality reduction, and 20 cell clusters were successfully classified. **(f)** Classification of cell clusters in each sample. **(g)** All 13 cell clusters in NSCLC were annotated with SingleR and CellMarker according to the composition of marker genes. **(h)** Expression levels of marker genes for each cell cluster.

Based on three datasets (GSE117570, GSE143423, GSE150660), we first conducted quality control on the RNA-seq data, resulting in 29,053 core cells. The three datasets exhibited comparable quantities of genes and transcripts (**Figure 1a**). Subsequently, we visualized the results by plotting variance based on differential gene expression and average expression levels of all NSCLC cells. A total of 14,042 highly variable genes and 2,000 non-variable genes were identified, with genes such as MALAT1, S100A6, HSPA1A, TAC3, CCL17, FGG, FTL, CD74, and TYROBP showing particularly significant expression differences between cells (**Figure 1b**). We then performed UMAP dimensionality reduction on all quality-controlled cells (n = 29,053), revealing distinct distribution patterns across the three datasets (**Figure 1c**). PCA-based clustering was applied to cell subpopulations, and the PCs were ranked by standard deviation. The first 20 PCs were retained, and the 20th PC showed a low standard deviation and significant statistical relevance (**Figure 1d**). UMAP further visualized the distribution of the 20 cell subpopulations (**Figure 1e**). Next, we annotated the subpopulations within each dataset, successfully distinguishing the PCs distributions (**Figure 1f**). Using the SingleR method, we annotated 13 major cell types, including B (259), CD4Tconv (1,932), CD8T (1,909), DC (1,149), Endothelial (440), Epithelial (501), Malignant (12,280), Mono/Macro (8,044), NK (864), Plasma (265), Oligodendrocyte (133), Pericytes (366), and CD8Tex (911) (**Figure 1g**). Ultimately, we conducted a comparison of the expression profiles of marker genes associated with these thirteen primary cell subpopulations, identifying significantly high expression of CXCR4, CD37, CREM, and TYROBP across multiple subpopulations (**Figure 1h**).

### 3.2 Investigating the metabolic profiles of individual cell subpopulations

**Figure 2:**
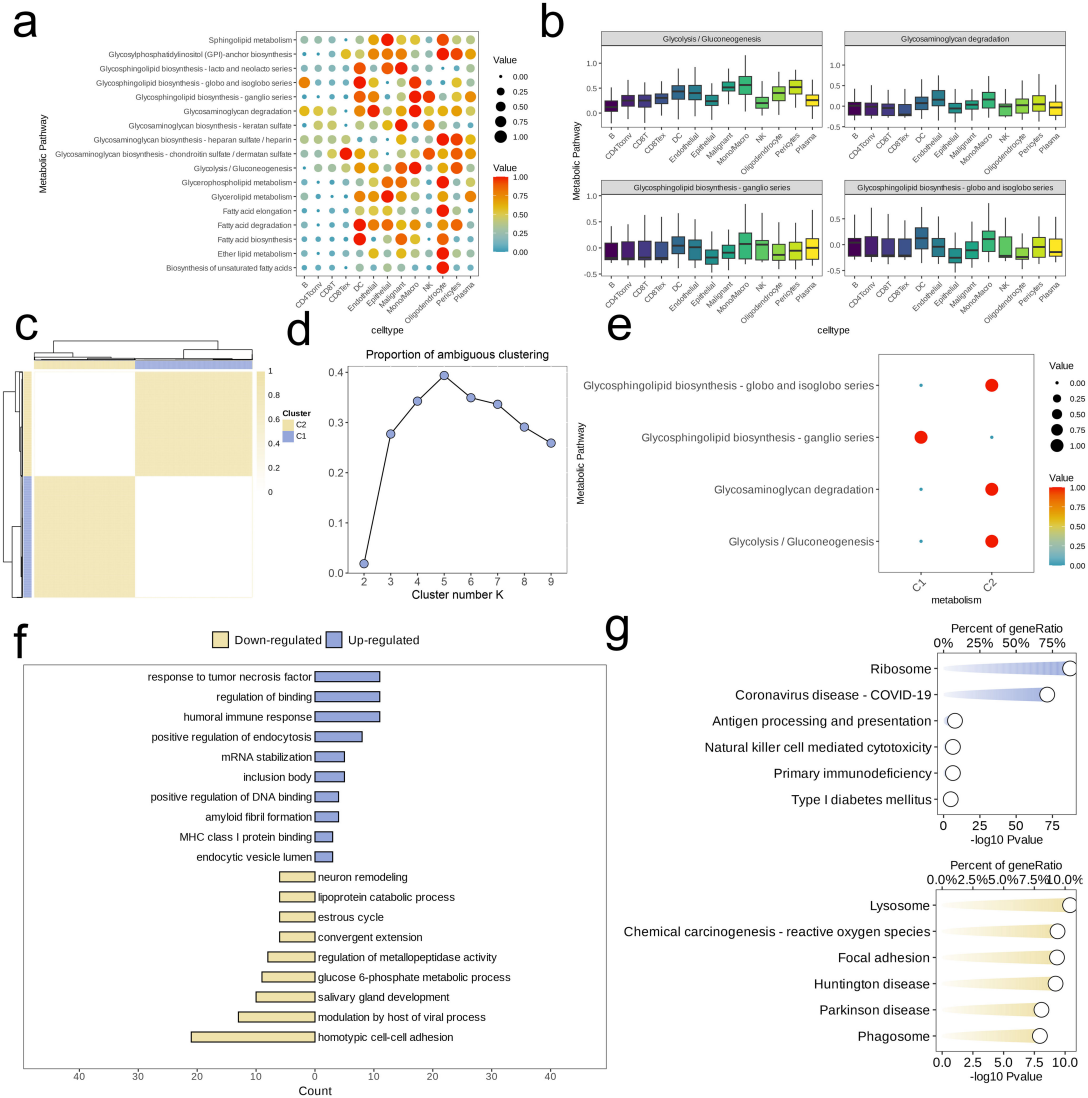
Identification of cell clusters with highly activated metabolism activities in NSCLC at the single cell level. **(a)** The highly activated metabolic process of in each cell cluster revealed by the scMetabolism R package. **(b)** Boxplots showing the activities of four highly activated metabolic pathways in malignant cells. **(c)** Heatmap of clustering at consensus k = 2. **(d)** PAC score, a low value of PAC implies a flat middle segment, allowing conjecture of the optimal k (k = 2) by the lowest PAC. **(e)** Two distinct metabolism patterns of NSCLC at the single-cell level unraveled by the unsupervised clustering. **(F, G)** Barplot reveals the dysregulated GO-BP terms **(f)** and KEGG pathways **(g)** in NSCLC cells with highly activated metabolism activities.

We analyzed the metabolic pathway activity across different cell subpopulations, revealing significant differences in pathway activation levels. Specifically, the Oligodendrocyte subpopulation exhibited the highest activity across metabolic pathways, while CD4Tconv and CD8T cells showed relatively lower metabolic activity. Among the Malignant cells, four pathways—Glycolysis/Gluconeogenesis, Glycosphingolipid biosynthesis-ganglio series, Glycosaminoglycan degradation and Glycosphingolipid biosynthesis-globo and isoglobo series—were highly activated (**Figure 2a**). Our attention was directed towards the activation status of these pathways among diverse cell subpopulations, and boxplot visualization showed that the Mono/Macro subpopulation generally exhibited higher activation levels than other subpopulations (**Figure 2b**).

Next, unsupervised clustering was conducted based on activity levels of these four pathways. Using the “high cohesion, low coupling” principle and PAC scoring, we selected k = 2 and identified two clusters (C1 and C2, **Figures 2c, d**). Assessing and comparing the degrees of activation of the four metabolic pathways amongst these clusters, we found that, except for the Glycosphingolipid biosynthesis-ganglio series, the metabolic activity in C2 was significantly higher than in C1 (**Figure 2e**). For C2, we identified high-expression genes and performed ORA enrichment analysis. The results indicated that these genes were predominantly concentrated within specific metabolic pathways related to cell signaling and immune response (e.g., response to tumor necrosis factor, humoral immune response, MHC class I protein binding), transcriptional regulation (positive regulation of DNA binding), endocytosis and intracellular transport (positive regulation of endocytosis, endocytic vesicle lumen), RNA and protein stability (mRNA stabilization), and protein folding and aggregation (inclusion body). However, pathways related to cell development and remodeling (e.g., neuron remodeling, salivary gland development, estrous cycle), substance metabolism (e.g., lipoprotein catabolic process, glucose 6-phosphate metabolic process), and cell-cell interaction (e.g., homotypic cell-cell adhesion, convergent extension) were downregulated in C2 (**Figure 2f**). Additionally, GSEA revealed upregulation in C2 for pathways associated with the Ribosome and Coronavirus disease-COVID-19, while pathways such as Lysosome, Chemical carcinogenesis-reactive oxygen species, Focal adhesion, Huntington disease, Parkinson disease, and Phagosome were relatively downregulated (**Figure 2g**).

### 3.3 Analysis of malignant cell subpopulations

**Figure 3:**
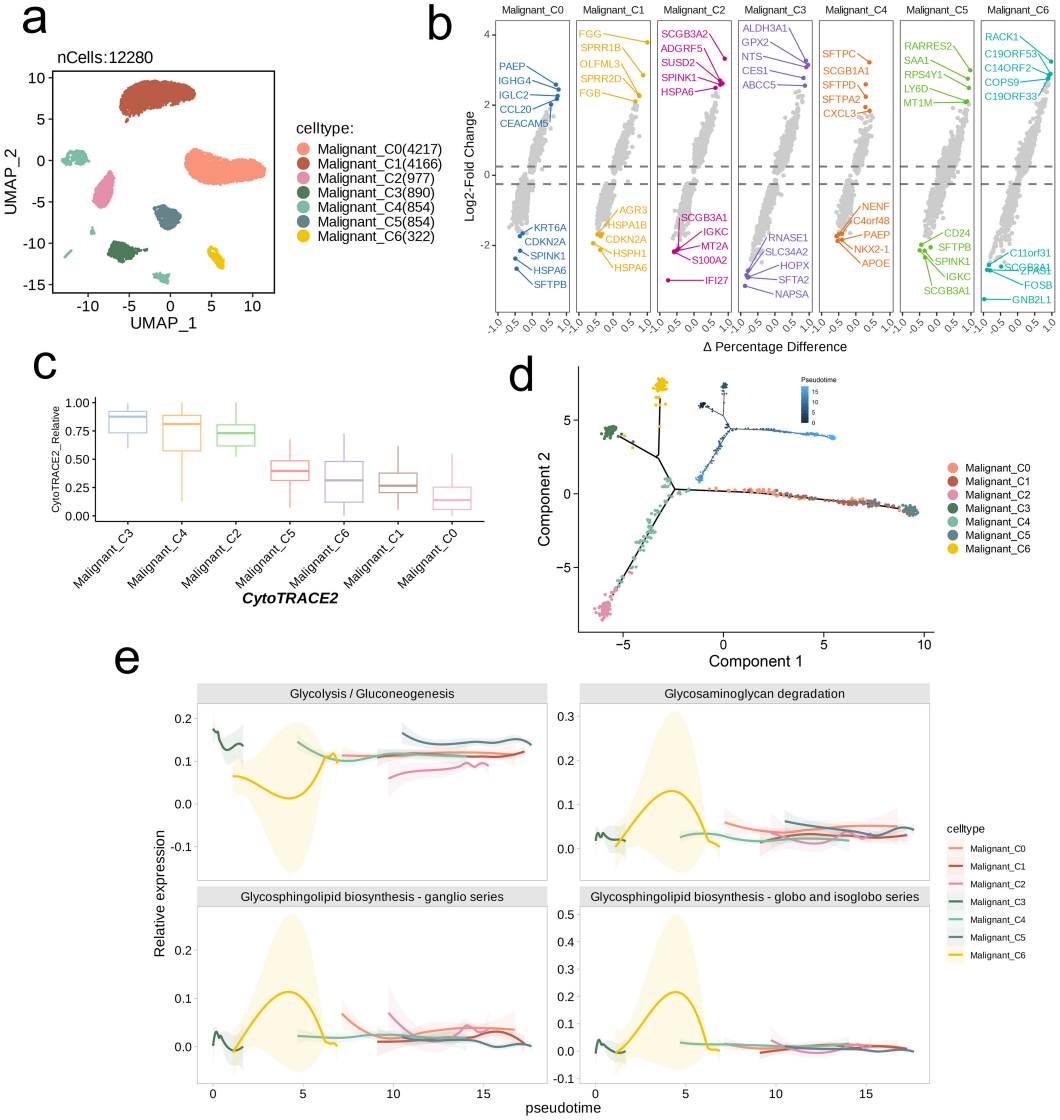
Trajectory analysis of NSCLC cell subsets with distinct differentiation patterns. **(a)** UMAP visualization of the subsets of malignant cells. **(b)** Volcano plots showing the celltype-specific markers of each subset. **(c)** Boxplots showing the predicted cellular potency and absolute developmental potential of malignant cell subset. **(d)** Trajectory analysis revealed cell subsets of malignant cells with distinct differentiation states. **(e)** The variations of metabolic pathway activities along with the pseudotime.

We selected a malignant cell subpopulation (n = 12,280) for further UMAP dimensionality reduction and clustering, which identified seven distinct subpopulations (**Figure 3a**). We analyzed the gene expression levels in each subpopulation, identifying marker genes, and visualized the differential expression of genes using a volcano plot. The top five downregulated and upregulated genes in each subpopulation were annotated in the plot as subpopulation-specific markers (**Figure 3b**). Using CytoTRACE2, we predicted the absolute developmental potential of the malignant cell subpopulations. The results showed that Malignant_C3 had the highest differentiation potential, followed by Malignant_C4 and Malignant_C2, which also exhibited relatively high differentiation potential. In contrast, Malignant_C5, Malignant_C6, Malignant_C1, and Malignant_C0 exhibited lower differentiation potential, with Malignant_C0 having the lowest potential (**Figure 3c**). Additionally, we performed pseudotime analysis, which revealed that Malignant_C3 was situated at the start of the developmental trajectory. As pseudotime increased, differentiation proceeded to Malignant_C6 and Malignant_C4, followed by Malignant_C0, Malignant_C2, and Malignant_C1, with Malignant_C5 occupied the final stage of the developmental trajectory (**Figure 3d**). The pseudotime differentiation points of the subpopulations corresponded with their differentiation potentials. The observations were consistent with the activity patterns of the four metabolic pathways across various subpopulations. Malignant_C3 and Malignant_C6 showed early and strong activation of these pathways, followed by Malignant_C4, Malignant_C0, Malignant_C1, and Malignant_C2, while Malignant_C5 displayed the latest stage of activation and the weakest intensity among the pathways. The Glycolysis/Gluconeogenesis pathway was overall more active than the other three pathways in all subpopulations (except Malignant_C6), with an inverse trend in activity compared to the other pathways. The lowest activity of this pathway was observed in Malignant_C6, whereas the peak activity of the other three pathways was seen in Malignant_C6. As pseudotime advanced, the activation patterns of Glycosphingolipid biosynthesis-ganglio series, Glycosaminoglycan degradation, and Glycosphingolipid biosynthesis-globo and isoglobo series exhibited greater similarity among the subpopulations (**Figure 3e**).

### 3.4 Analysis of differential expression and pathway enrichment

**Figure 4:**
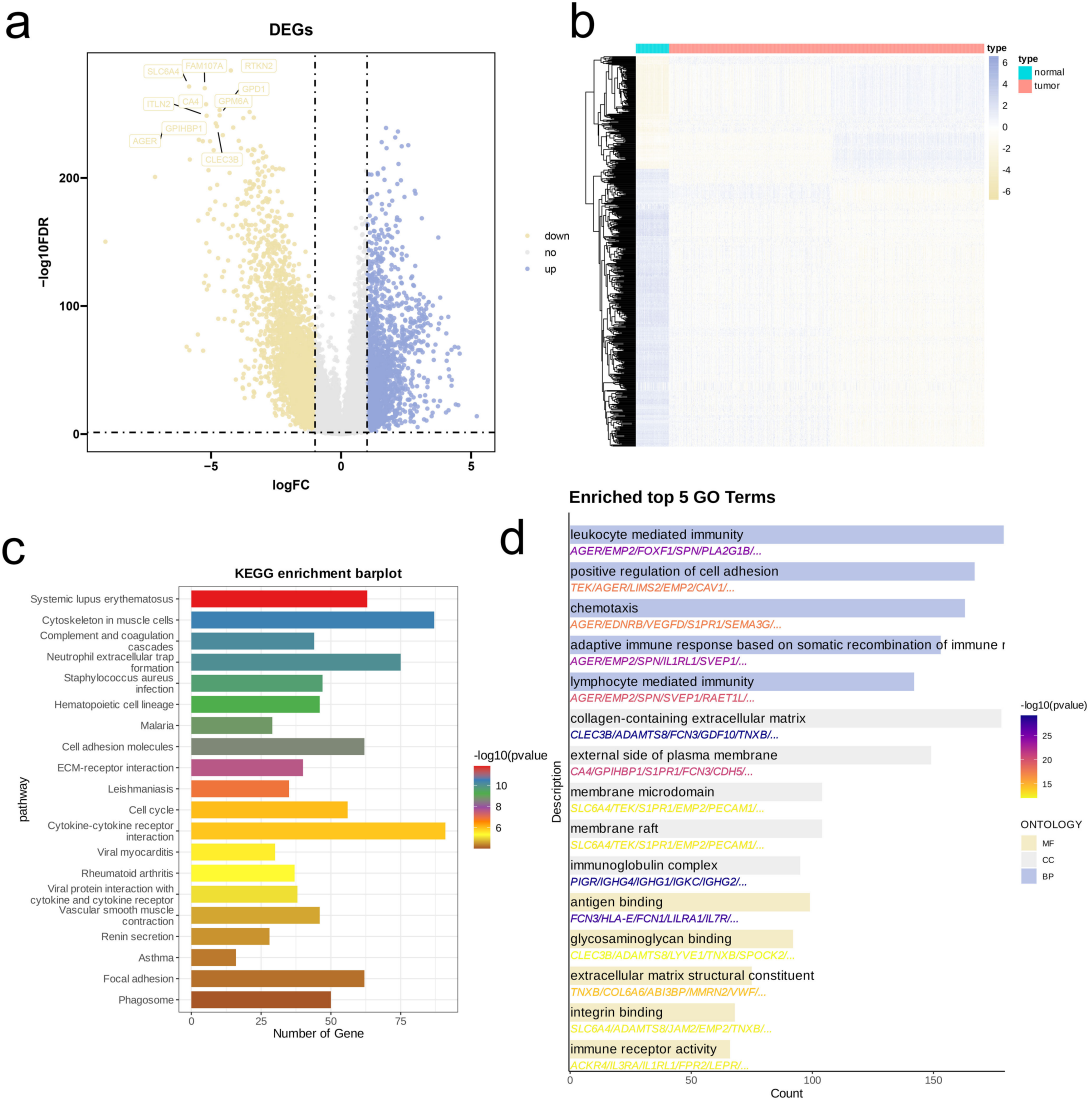
Identification and functional enrichment analysis of DEGs between NSCLC patients and controls. **(a)** Volcano plot of DEGs between OS and control in the merged cohort of TCGA-LUAD and TCGA-LUSC. p < 0.05 and |log_2_FoldChange|>1 was identified as significant DEGs. **(b)** Heatmap of DEGs. **(c, d)** KEGG pathways **(c)**, and barplots of the BP, CC, MF **(d)** of DEGs.

Using a combined bulk RNA-seq dataset (TCGA-LUAD, TCGA-LUSC), we first performed differential expression analysis. Volcano plots and heatmaps revealed that, compared to tumor tissues, normal tissues exhibited a higher number of genes with elevated expression levels (**Figures 4a, b**). Next, we performed KEGG pathway analysis on DEGs. As illustrated by bar plots, the enrichment of DEGs was predominantly highlighted in pathways related to immune responses and inflammation (e.g., Systemic lupus erythematosus, Neutrophil extracellular trap formation, Cytokine-cytokine receptor interaction), cell structure and motility pathways (e.g., Cytoskeleton in muscle cells, Cell adhesion molecules, Focal adhesion) (**Figure 4c**). We also conducted GO analysis and visualized top five enriched pathways in BP, CC, and MF. Similar to KEGG results, DEGs were considerably enriched in immune-related pathways (e.g., Leukocyte mediated immunity, Lymphocyte mediated immunity, Antigen binding, Immune receptor activity), cell adhesion and migration pathways (e.g., Positive regulation of cell adhesion, Chemotaxis, Integrin binding), and extracellular matrix and membrane-related pathways (e.g., Collagen-containing extracellular matrix, Extracellular matrix structural constituent, Glycosaminoglycan binding) (**Figure 4d**).

### 3.5 WGCNA identification of metabolism-related hub genes

**Figure 5:**
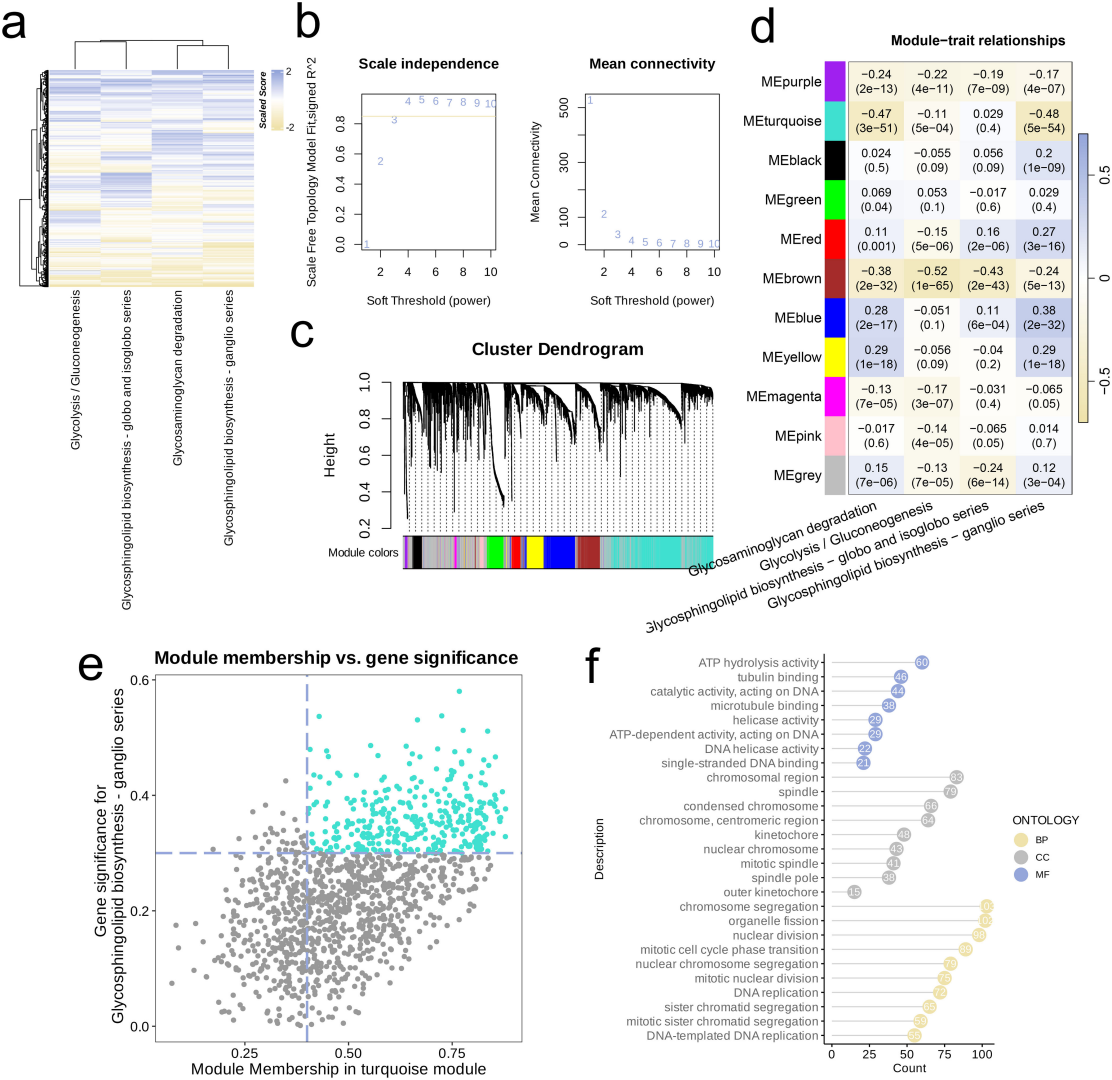
Metabolism-related genes were screened by WGCNA. **(a)** Heatmap of the four highly activated metabolic pathways. **(b)** Analysis of the scale-free index for various soft-threshold powers (β). **(c)** Cluster dendrogram of the co-expression modules. Each color indicates a co-expression module. **(d)** Module-trait heatmap displaying the correlation between module eigengenes and clinical traits. **(e)** Correlation between module membership and gene significance in the turquoise modules. Dots in colors were regarded as the hub genes of the module. **(f)** The top enriched GO terms of the hub genes of the module.

We investigated activation levels of four metabolic pathways in the combined TCGA-LUAD and TCGA-LUSC cohort using GSVA scoring, visualized with a heatmap. Overall, these four metabolic pathways were upregulated in most samples (**Figure 5a**). We then performed network topology analysis, selecting β = 3 as the optimal soft threshold determined by scale independence and mean connectivity, which resulted in a network with favorable scale-free characteristics and moderate sparsity (**Figure 5b**). We constructed a hierarchical clustering dendrogram of co-expression modules, identifying ten distinct modules (**Figure 5c**). Following this, we calculated the correlations among the ten modules and the four metabolic pathways, revealing a significant negative association between the MEturquoise module and three of these pathways (R < -0.1, p < 0.05, **Figure 5d**). Genes from the MEturquoise module were then selected for further screening, leading to the identification of hub genes (**Figure 5e**). Finally, analysis of GO of these hub genes demonstrated that they were predominantly enriched in DNA binding and hydrolysis-related activities (e.g., ATP hydrolysis activity, Catalytic activity acting on DNA, ATP-dependent DNA helicase activity, Single-stranded DNA binding), cytoskeleton-related pathways (e.g., Tubulin binding), cell cycle and division-related pathways (e.g., Mitotic cell cycle phase transition, Nuclear division, Chromosome segregation), and DNA replication pathways (e.g., DNA replication, DNA-templated DNA replication) (**Figure 5f**).

### 3.6 Developing a risk signature for NSCLC patients utilizing the TCGA combined cohort

**Figure 6:**
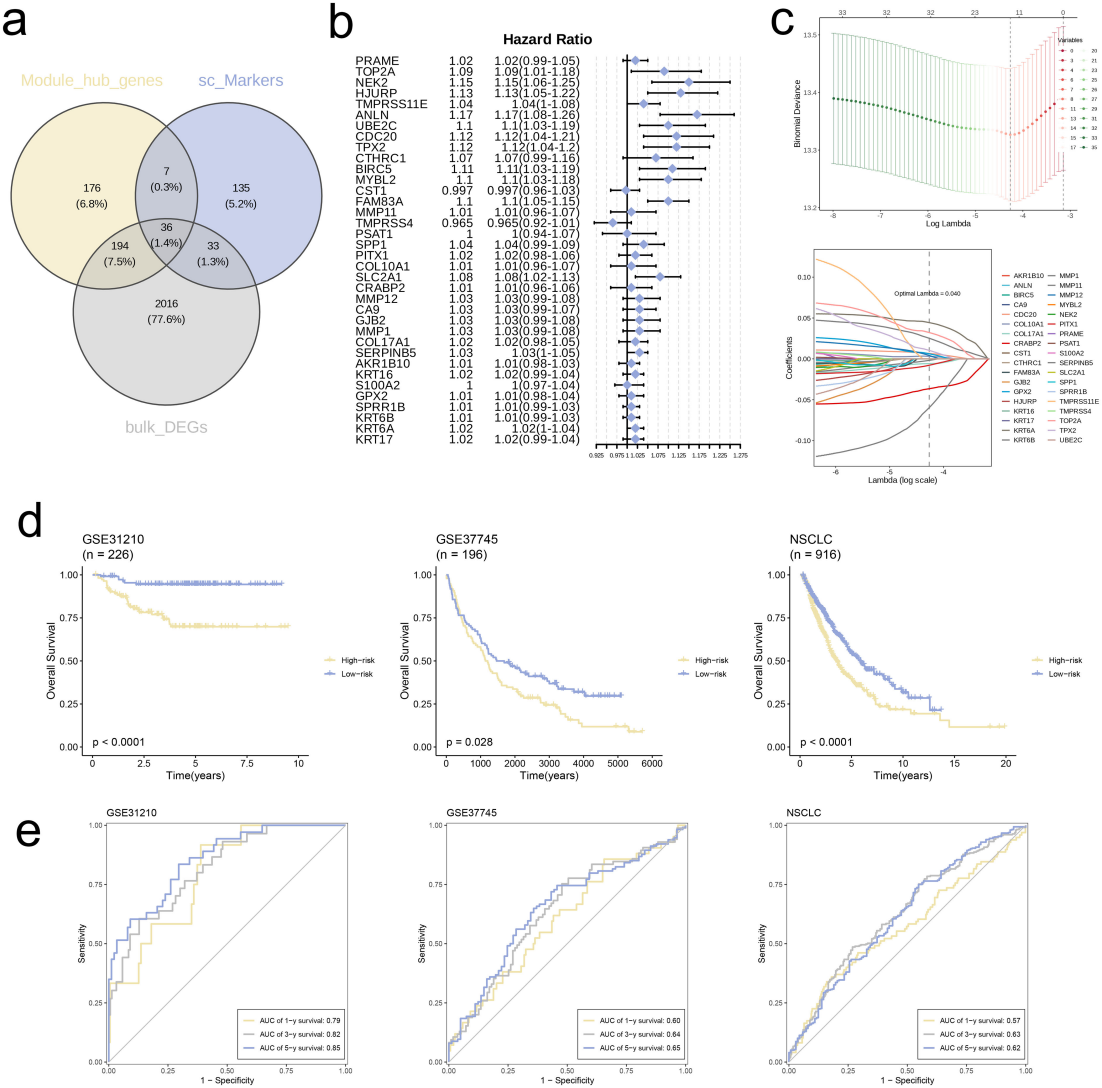
Construction of risk signature in the merged TCGA cohort. **(a)** Venn diagram analysis of hub genes of modules, single-cell markers, and DEGs from the merged TCGA cohort. **(b)** Univariate cox regression analysis of 36 genes in the merged TCGA cohort. **(c)** The selection of prognostic genes based on the optimal parameter λ that was obtained in the LASSO regression analysis. **(d)** K-M curves displayed survival outcomes of patients in two risk groups from the three cohorts. **(e)** Time-dependent ROC curves were drawn to assess survival rate at 1-year, 3-year, and 5-year in the three cohort.


First, through the overlapping process of hub genes identified via WGCNA analysis, marker genes from single-cell analysis, and DEGs, we obtained a collection of 36 genes, which was depicted in a Venn diagram (**Figure 6a**). We conducted Cox regression analysis on these genes and found that several were associated with poor prognosis in NSCLC (**Figure 6b**). Also, we executed analysis of LASSO regression on intersecting genes (**Figure 6c**), selecting optimal lambda value of 0.040 to refine the prognostic gene set. Through cross-validation curves and coefficient path distributions, we identified 13 genes with corresponding coefficients, yielding the following risk score formula:

$$\begin{array}{c} \text{Risk score }=\text{ TOP}2\text{A }*\;\left(0.05\right)\;+\text{ CDC}20\;*\;\left(0.013\right)\\ \;+\text{ TPX}2\;*\;\left(0.03\right)\;+\text{ MMP}11\;*\;\left(-0.012\right)\\ \;+\text{ PITX}1\;*\;\left(-0.022\right)\;+\text{ COL}10\text{A}1\;*\;\left(0.007\right)\\ \;+\text{ CRABP}2\;*\;\left(-0.047\right)\;+\text{ MMP}12\;*\;\left(0.024\right)\\ \;+\text{ MMP}1\;*\;\left(-0.124\right)\;+\text{ COL}17\text{A}1\;*\;\left(-0.007\right)\\ \;+\text{ GPX}2\;*\;\left(0.019\right)\;+\text{ KRT}6\text{B }*\;\left(0.047\right)\\ \;+\text{ KRT}6\text{A }*\;\left(0.057\right) \end{array}$$

We used TCGA combined cohort as training set and validated model using datasets GSE31210 and GSE37745. KM survival curves for all three cohorts revealed significantly worse prognosis for high-risk group in comparison with low-risk group (p < 0.05, **Figure 6d**). Furthermore, ROC curves for 1-, 3-, and 5-year survival rates in all three independent cohorts demonstrated that the model exhibited good predictive performance (AUC > 0.55, **Figure 6e**).

### 3.7 Correlation analysis based on risk signature

**Figure 7:**
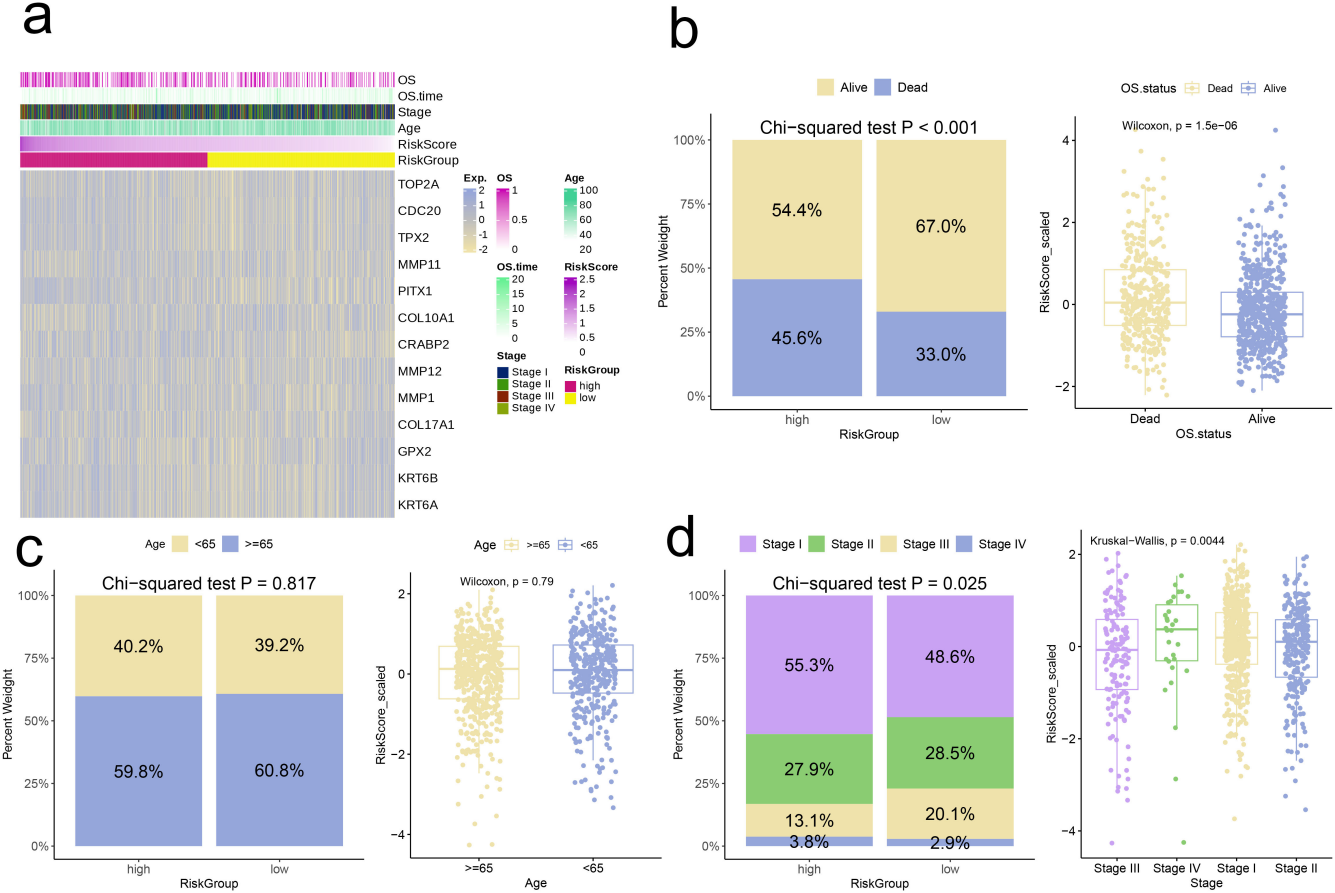
Correlation analysis of risk scores with clinical characteristics. **(a)** Heatmap of risk model and clinical characteristics. **(b–d)** Relationship between age, stage, and survival status with the analysis model.

Further analyses were conducted, categorizing individuals into high-risk and low-risk groups based on their respective Risk scores. At the outset, we analyzed and contrasted the expression variations of signature genes and clinical attributes between the two risk groups, subsequently presenting the findings through a heatmap visualization (**Figure 7a**). The comparison showed that a greater proportion of patients in high-risk group had deceased, and Risk scores of deceased patients were notably higher compared to those of surviving patients (p < 0.001, **Figure 7b**). Conversely, the Risk scores and patient proportions among various age groups did not exhibit any statistically significant differences (Age < 65 and Age ≥ 65, p > 0.05, **Figure 7c**). Additionally, the high-risk group comprised a larger percentage of Stage IV patients in comparison to the low-risk group (p = 0.025). Moreover, the Risk scores for patients in Stage IV were markedly elevated compared to those in Stages I, II, and III (p = 0.0044, **Figure 7d**).

**Figure 8:**
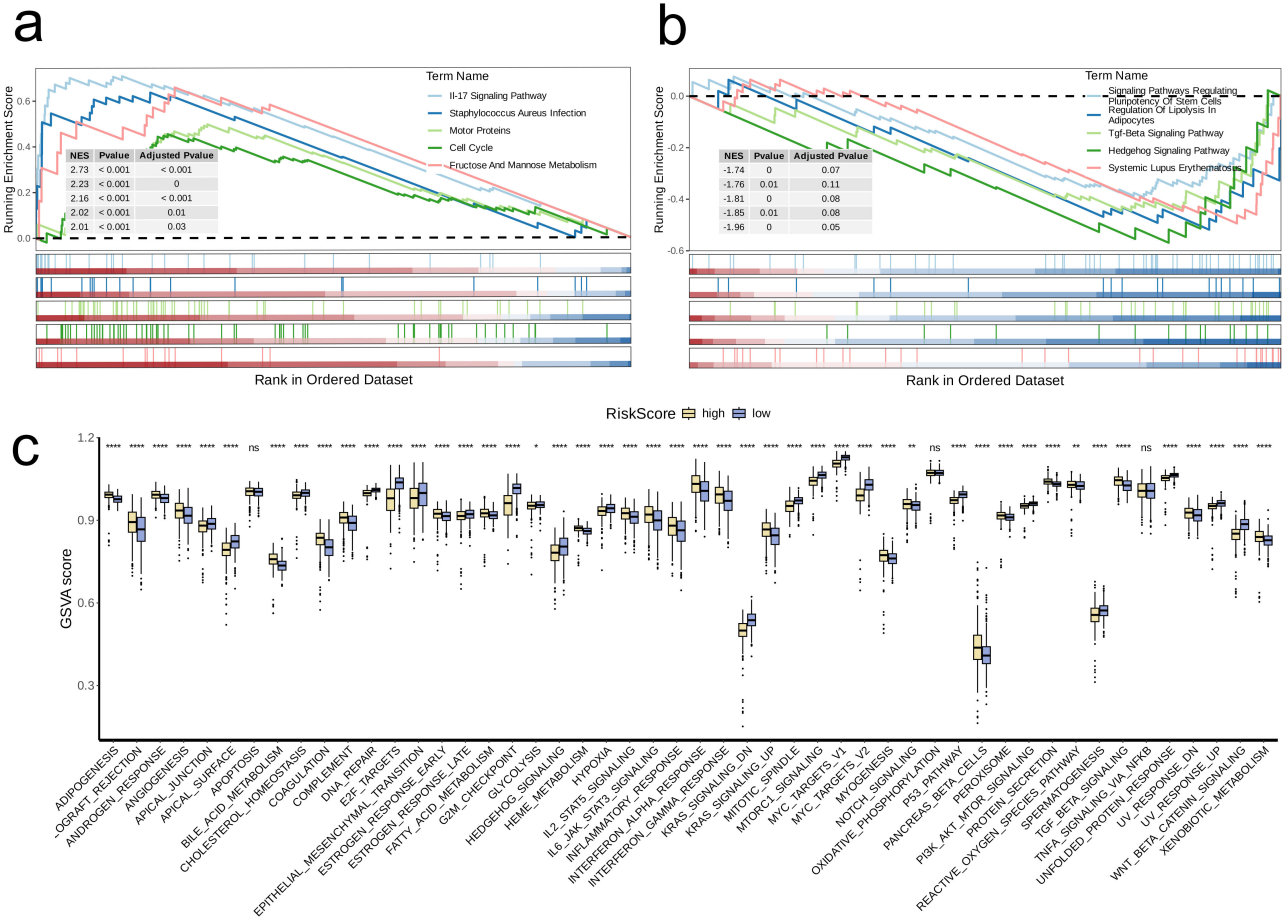
Biological characteristics between high-and low-risk groups. **(a, b)** The upregulated **(a)** and downregulated **(b)** KEGG pathways in high-risk group. **(c)** The differences of estimated GSVA scores of cancer hallmarks between high- and low-risk groups. A p value < 0.05 was considered statistically significant (* p < 0.05; ** p < 0.01; **** p<0.0001; ns, no significance).

Next, we conducted GSEA based on DEGs in the high-risk group. The findings revealed a notable increase in the expression of multiple pathways within the high-risk cohort, including IL-17 Signaling Pathway, Staphylococcus Aureus Infection, Motor Proteins, Cell Cycle, and Fructose and Mannose Metabolism (adjusted p-value < 0.05, **Figure 8a**). Conversely, Systemic Lupus Erythematosus showed significant upregulation in the low-risk group (adjusted p-value < 0.05, **Figure 8b**).

We further GSVA to compare the scores of different cancer hallmark pathways between the two risk groups. The analysis indicated that high-risk group had significantly lower GSVA scores than low-risk group for following pathways: APICAL_JUNCTION, CHOLESTEROL_HOMEOSTASIS, APICAL_SURFACE, EPITHELIAL_MESENCHYMAL_TRANSITION, DNA_REPAIR, E2F_TARGETS, ESTROGEN_RESPONSE_LATE, G2M_CHECKPOINT, HEDGEHOG_SIGNALING, HYPOXIA, MTORC1_SIGNALING, MYC_TARGETS_V1, KRAS_SIGNALING_DN, MITOTIC_SPINDLE, MYC_TARGETS_V2, P53_PATHWAY, SPERMATOGENESIS, UNFOLDED_PROTEIN_RESPONSE, PI3K_AKT_MTOR_SIGNALING, UV_RESPONSE_UP, and WNT_BETA_CATENIN_SIGNALING (p < 0.05, **Figure 8c**).

**Figure 9:**
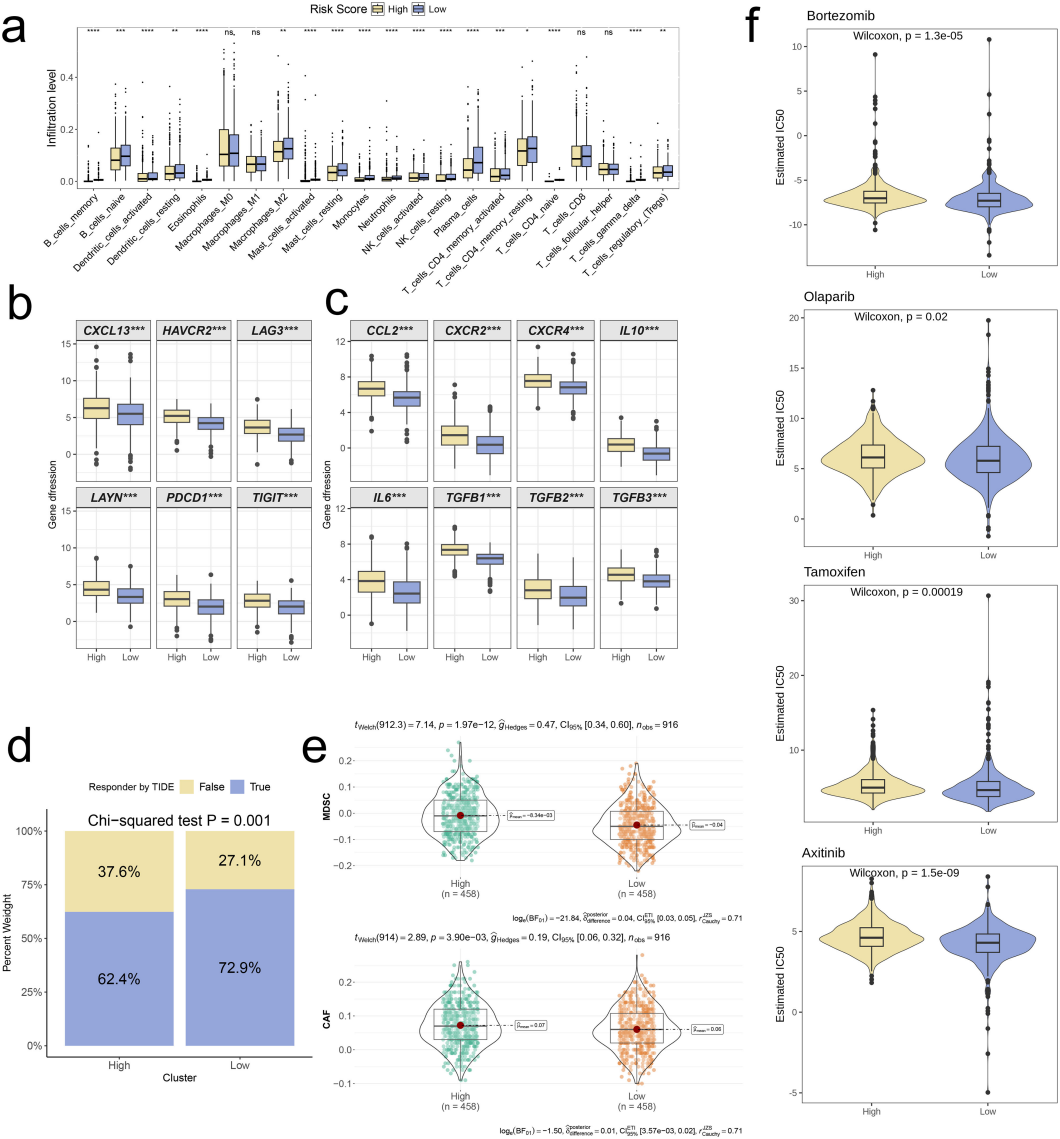
Distinct TME landscapes and therapeutic agents between high-and low-risk groups. **(a)** Box plot illustrating the distributions of 22 immune cell subsets determined by CIBERSORT between two risk groups. **(b, c)** Box plot illustrating the expression profiles of T cell exhaustion markers **(b)** and M2 polarization regulators **(c)** between two risk groups. **(d)** Stacked plot showed the distribution of predicted responders determined by the TIDE webtool between two risk groups. **(e)** Violin plot displaying the infiltration levels of CAF and MDSC between two risk groups. **(f)** Violin plot displaying the estimated IC50 of therapeutic agents between two risk groups. A p value < 0.05 was considered statistically significant (* p < 0.05; ** p < 0.01; *** p < 0.001; **** p<0.0001; ns, no significance).

Following that, we applied the CIBERSORT algorithm to quantify the infiltration levels of 22 immune cell types in both risk groups. Among the results that were statistically significant, the low-risk group exhibited significantly elevated levels of immune cell infiltration in comparison to the high-risk group (p < 0.05, **Figure 9a**). Boxplot analysis further revealed that high-risk group had significantly higher expression levels of T cell exhaustion markers (HAVCR2, CXCL13, LAYN, LAG3, PDCD1, TIGIT) and M2 polarization regulators (CXCR2, CCL2, IL10, CXCR4, IL6, TGFB2, TGFB1, TGFB3) In contrast to the low-risk group (p < 0.001, **Figures 9b, c**).

To further investigate distinctions in immune evasion capabilities between risk groups, we assessed TIDE scores. The stacked plot revealed a notably smaller proportion of “True” responses in the high-risk group, in comparison to the low-risk group (p = 0.001, **Figure 9d**). Additionally, the violin plot showed that infiltration levels of MDSC and CAF were significantly higher in high-risk group in comparison with low-risk group (p < 0.001, **Figure 9e**). Ultimately, we conducted a drug sensitivity prediction using Inhibitory Concentration 50 (IC50) values, observing that patients belonging to the high-risk group demonstrated reduced sensitivity to Bortezomib, Olaparib, Tamoxifen, and Axitinib when compared to those in the low-risk group (p < 0.05, **Figure 9f**).

### 3.8 KRT6B enhances tumorigenic potential in lung cancer cells

**Figure 10:**
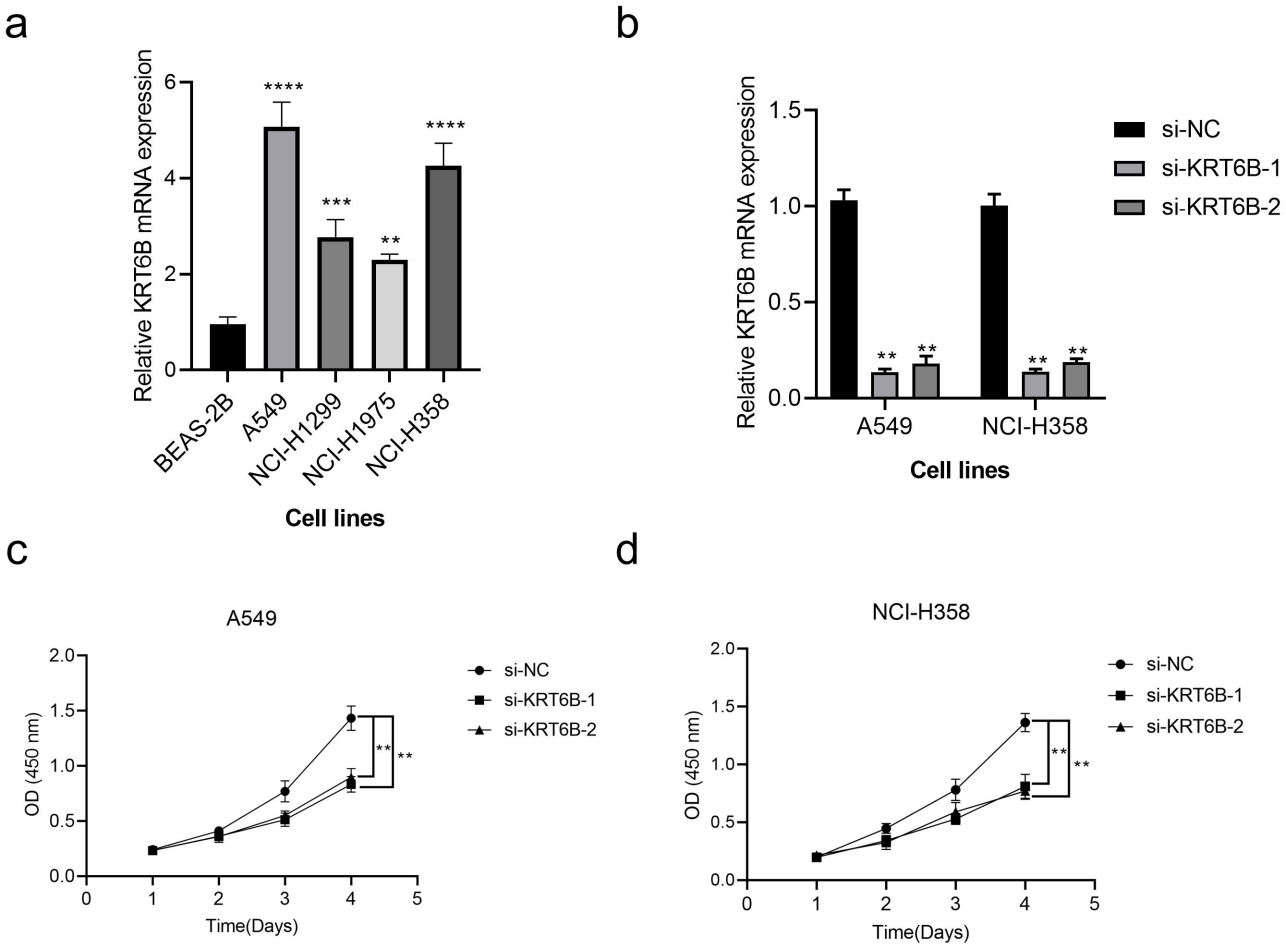
*In vitro* validation of the role of KRT6B in NSCLC. **(a)** RT-qPCR analysis shows high expression of KRT6B in cancer cells. **(b)** RT-qPCR results confirm that both si-KRT6B constructs effectively knock down KRT6B expression. **(c)** CCK8 assay demonstrates that KRT6B knockdown significantly inhibits the proliferation of A549 cells. **(d)** CCK8 assay shows that KRT6B knockdown significantly inhibits the proliferation of NCI-H358 cells. A p value < 0.05 was considered statistically significant (** p < 0.01; *** p < 0.001; **** p<0.0001).

To evaluate the variation in KRT6B mRNA expression among five cell lines, we initiated the process by conducting RT-qPCR. Subsequently, we used siRNA-mediated knockdown of KRT6B expression in the A549 and NCI-H358 lung cancer cell lines and conducted CCK8 assays. The results obtained from RT-qPCR indicated that the expression of KRT6B was markedly elevated in four lung cancer cell lines, in contrast to the normal BEAS-2B cell line (p < 0.01, **Figure 10a**). To validate the knockdown efficiency, we confirmed that both siRNAs effectively reduced KRT6B expression (p < 0.01, **Figure 10b**). The results of the CCK8 assay showed a notable reduction in absorbance for both cell lines when KRT6B was knocked down, in comparison to the control groups, indicating that KRT6B promotes lung cancer cell proliferation (p < 0.01, **Figures 10c, d**).

## 4 Discussion

Globally, lung cancer holds the distinction of being the most common malignancy and persists as the leading cause of cancer-related deaths among both males and females (16). NSCLC accounts for roughly 85% of all lung cancer instances (17). The etiological factors for NSCLC include smoking, secondhand smoke, air pollution, genetic predisposition, and other environmental influences. Despite advancements in early detection and treatment, the five-year survival rate for NSCLC remains below 20% (18). Recently, targeted therapies have gained traction, but only a small proportion of patients benefit, and the development of resistance is common. Recent studies indicate that metabolic reprogramming is crucial for cancer progression. Tumor cells typically exhibit the “Warburg effect”, where they preferentially rely on glycolysis for energy production, even under aerobic conditions (19). This metabolic shift supports rapid tumor proliferation and results in the accumulation of lactate, which acidifies the TME and promotes the development of drug resistance. Furthermore, the degradation products of (GAGs have been shown to influence tumor invasion and metastasis. Additionally, glycosphingolipids play important roles in cell signaling and recognition, with aberrant expression of gangliosides closely linked to the invasiveness and drug resistance of NSCLC. Therefore, a deeper understanding of the regulatory mechanisms governing these metabolic pathways and their activation in NSCLC cells could not only offer insights into the progression of NSCLC but also recognize potential therapeutic targets for future treatment approaches.

For the current study, we sourced bulk RNA-seq, scRNA-seq data, along with clinical information pertaining to NSCLC, from various databases including TCGA, GEO, and TISCH2. We first identified genes with significant differential expression across various cell types in single-cell dataset. The results revealed that genes such as MALAT1, S100A6, HSPA1A, TAC3, CCL17, FGG, FTL, CD74, and TYROBP exhibited notable expression differences between cell types. These expression heterogeneities may reflect the roles of these genes in the onset and progression of NSCLC, providing valuable clues for subsequent mechanistic investigations. Next, we annotated the cell subpopulations and successfully determined 13 major cell types. A comparison between the distribution of the top 20 PCs and the 13 identified cell types indicated that some subpopulations might warrant further subdivision. These cell type annotations offer valuable insights into the TME of NSCLC. Notably, after comparing the expression levels of marker genes across these subpopulations, our findings showed that the CREM gene was highly expressed in several cell types. Established as a crucial transcriptional regulator, CREM is involved in numerous physiological processes including the control of the cell cycle, the response to stress, and the modulation of the immune system (20). The high expression of CREM in different cell subpopulations suggests that it may play multifaceted roles in NSCLC.

Subsequently, we performed a metabolic analysis involving the 13 primary cell types to investigate the metabolic diversity present among various cell populations. Our findings revealed that four specific metabolic pathways—Glycolysis/Gluconeogenesis, Glycosphingolipid biosynthesis (ganglio series), Glycosaminoglycan degradation, and Glycosphingolipid biosynthesis (globo and isoglobo series)—were significantly activated in cancerous cell subpopulations. These changes in metabolism indicate the capacity of tumor cells to adjust to their surroundings. Moreover, we hypothesize that the heightened activity of these pathways is linked to the tumor-specific metabolic reprogramming, which supplies the energy and support necessary for cancer cells’ rapid proliferation, invasion, and metastasis. Furthermore, we noted activation of these four pathways was generally higher in the Mono/Macro cell subpopulations compared to other subtypes. Given that Mono/Macro cells are typically involved in immune regulation, these metabolic changes may relate to the functional status of immune cells, such as promoting inflammatory responses or immune suppression, which may, in turn, facilitate immune escape and tumor progression. In addition, the discovery of the highly activated state of four key metabolic pathways in malignant cells provides new insights into the metabolic characteristics of NSCLC. First, with respect to treatment resistance, we observed that malignant cells support their rapid proliferation and survival by enhancing metabolic pathways such as glycolysis/gluconeogenesis, which may explain why some traditional therapies are less effective (21, 22). For example, lactic acid accumulation leads to acidification of the tumor microenvironment, promoting the development of drug resistance (23). Secondly, in terms of immune escape, changes in metabolic activity affect the infiltration and function of immune cells, such as the high activation of metabolic pathways in mononuclear/macrophage subsets may lead to the formation of immunosuppressive microenvironments.

In addition, although the current targeted therapy has a certain effect, the proportion of patients who benefit is low and prone to drug resistance. In contrast, risk models based on metabolic characteristics can more accurately identify high-risk patient groups and provide a basis for personalized treatment. Combined with immune checkpoint inhibitors, interventions targeting specific metabolic pathways may reverse immunosuppressive states and improve treatment response rates. For example, modulating glycolysis levels or altering specific lipid metabolic pathways may enhance T cell activity, resulting in synergies with immunotherapy. In-depth understanding of metabolic reprogramming in NSCLC not only helps to reveal the mechanism of disease progression, but also provides a theoretical basis for developing new combination treatment strategies.

Through unsupervised clustering analysis, we identified two distinct groups (C1 and C2), with C2 showing significantly higher activation in three metabolic pathways, excluding Glycosphingolipid biosynthesis-ganglio series, compared to C1. This suggests that the two groups exhibit notable differences in their metabolic profiles, with C2 displaying higher metabolic activity. Additional enrichment analysis using ORA showed that genes with high expression in C2 are predominantly associated with various pathways and biological processes related to cellular signal transduction, immune response, transcriptional regulation, endocytosis and intracellular transport, RNA and protein stability, and protein folding and aggregation. In contrast, C2 showed relatively lower gene enrichment in pathways such as cell development and remodeling, substance metabolism, and cell-cell interactions, indicating a less active state in these processes. GSEA results further corroborated our conclusions. We propose that C2 cells may possess enhanced protein synthesis capabilities and exhibit metabolic reprogramming, making them more adaptable and responsive to the TME’s metabolic demands and immune responses, while showing reduced activity in processes like phagocytosis and cell adhesion. Overall, our findings highlight significant metabolic heterogeneity between different cell subpopulations, providing new perspectives for understanding the metabolic processes in NSCLC and their impact on disease progression, and offering potential avenues for the development of prognostic therapeutic strategies in NSCLC.

Next, we focused on the Malignant cell subpopulations for targeted analysis. Using UMAP dimensionality reduction and clustering, we identified seven subgroups and revealed gene expression differences among them. These subgroups exhibit considerable diversity in gene expression patterns, with each subgroup displaying unique marker genes, indicating significant functional and expression differences even within the same Malignant cell category. The analysis conducted using CytoTRACE2 and pseudotime revealed substantial differences in the differentiation potential and developmental pathways among the subpopulations. Moreover, the activity of four metabolic pathways in different subgroups was generally consistent with their positions on the differentiation trajectory. In summary, Malignant_C3, positioned at the starting point of the developmental pathway, exhibits the strongest differentiation potential and high metabolic activity; Malignant_C6, at an early stage of differentiation, shows considerable fluctuations in metabolic pathway activity; following the differentiation of Malignant_C4, which has high differentiation potential, into Malignant_C0, Malignant_C2, and Malignant_C1, the changes in metabolic pathway activity become more stable; and Malignant_C5, potentially representing the terminal state of the differentiation trajectory, still retains some differentiation potential and metabolic activity. These dynamic changes in metabolic characteristics are closely linked to cell differentiation pathways, highlighting the complex cellular heterogeneity within Malignant cells and offering potential therapeutic targets and novel strategies for NSCLC treatment.

In differential expression and enrichment analysis, KEGG and GO enrichment results for DEGs revealed significant enrichment in immune system and inflammation-related pathways, cell adhesion and migration, and extracellular matrix in tumor tissues. These findings indicate that changes in the immune microenvironment might be critical for the progression of NSCLC. Specifically, the activation of immune responses and inflammation could be key mechanisms that allow tumor cells to evade immune surveillance and drive tumor progression.

Based on the GSVA scores derived from the activation levels of four metabolic pathways in the combined cohort, we successfully constructed a scale-free network and identified 10 co-expression modules by using WGCNA analysis. Upon observation, a notable inverse relationship was observed between the MEturquoise module and three distinct metabolic pathways in our analysis. GO analysis further revealed that the hub genes within the MEturquoise module were primarily enriched in pathways and biological processes related to cell cycle regulation, DNA replication, and maintenance of genomic stability. These recommendations indicate that these hub genes might be essential for tumor cell proliferation and survival, and could potentially serve as biomarkers or therapeutic targets for NSCLC in the future.

Based on these discoveries, we developed a risk signature utilizing data from the merged cohort. By employing Cox regression and LASSO regression analyses, we identified 13 prognostic genes in the model: TOP2A, CDC20, TPX2, MMP11, PITX1, COL10A1, CRABP2, MMP12, MMP1, COL17A1, GPX2, KRT6B, and KRT6A. TOP2A encodes a protein that activates the activity of type II DNA topoisomerase and plays a pro-cancer role in NSCLC (24). CDC20 is a positive regulator of cell division, and previous studies have shown its elevated expression in lung adenocarcinoma (25). The gene TPX2 encodes a microtubule-associated protein and has been established as both a diagnostic and prognostic indicator in various cancers (26). MMP11 encodes a secreted protein that regulates multiple physiological processes and signaling pathways, influencing cell behavior and playing a key role in the TME (27). The PITX1 gene, belonging to the PITX family, is crucial for normal embryonic development. Studies have shown that PITX family members exhibit overexpression in NSCLC compared to healthy lung tissue (28). The upregulation of COL10A1, which is a distinct cleavage product of type X collagen, is prominent in malignant tumors and holds a pivotal role in the development and advancement of tumors (29). CRABP2 encodes a special carrier for retinoic acid (RA), and its expression is notably elevated in NSCLC tissues relative to adjacent normal lung tissue (30). MMP12 encodes a matrix metalloproteinase and serves as an immune cell-related biomarker for squamous cell carcinoma of the lung, reflecting disease status and prognosis (31). MMP1, a member of the same gene family as MMP12, is considered an adverse prognostic factor in NSCLC (32). COL17A1 encodes the α chain of type XVII collagen and promotes cell proliferation in cancer tissues (33). GPX2, an enzyme belonging to the glutathione peroxidase group, protects cells from oxidative damage by converting hydrogen peroxide and fatty acid hydroperoxides into less reactive forms (34). KRT6B and KRT6A, members of the keratin gene family, are closely associated with clinical stage, tumor infiltration, and metastasis in patients (35, 36). The survival analysis using the Kaplan-Meier method revealed a notably worse outcome for the high-risk group in contrast to the low-risk group, highlighting the robust predictive stability of our model across three validation cohorts. Additionally, the model exhibited excellent predictive performance, as evidenced by the ROC curves corresponding to 1-year, 3-year, and 5-year survival rates. Our study offers a crucial foundation for evaluating prognosis and tailoring treatment plans for patients with NSCLC.

To further validate the clinical relevance of the risk signature model, we performed extensive biological and immunological investigations. Comparing the two groups, the proportion of deceased patients was significantly higher in the high-risk group compared to the low-risk group, and the risk scores of deceased patients were markedly elevated compared to survivors. Examination of clinical stage distribution showed a greater percentage of Stage IV patients within the high-risk group, with Stage IV patients exhibiting notably higher risk scores than those in other stages. This suggests that our model has potential for predicting patient prognosis and staging, offering strong support for clinical decision-making. Subsequently, we performed GSEA, which revealed that significant upregulation of the IL-17 signaling pathway was observed in high-risk group. The upregulation of the IL-17 pathway may reflect an enhanced inflammatory response in high-risk patients, which could be associated with immune responses and tumor progression in the TME (37). Furthermore, we used GSVA to compare the scoring of various cancer hallmark pathways between different risk groups. We found that the pathways in GSVA outcomes have in common the fact that they all influence cell fate decisions, viability, growth rate, differentiation state, and response to environmental changes to some extent. In the high-risk group, lower GSVA scores for these pathways indicate that their activity is suppressed, which may be a manifestation of dysregulation of intracellular mechanisms during tumor development or may reflect the effect of therapeutic interventions. They all point to a central question: how cells lose their normal regulation and turn into a malignant phenotype.

The CIBERSORT algorithm revealed that immune cell infiltration levels in low-risk group were generally higher than those in high-risk group. This suggests that stronger tumor immunity is potentially a key factor contributing to better prognosis in low-risk patients. Moreover, expression levels of T cell exhaustion markers and M2 polarization regulators were significantly higher in the high-risk group, indicating a more severe immunosuppressive state that could promote tumor progression (38). This may be one of the reasons for the poorer prognosis observed in the high-risk group. Further TIDE analysis confirmed that tumors from high-risk patients were more prone to evading immune surveillance. The high-risk group demonstrated notably increased infiltration levels of MDSCs and CAFs in comparison to the low-risk group. Frequently linked to the formation of an immunosuppressive microenvironment, the significance of these cell types emphasizes the crucial function of the TME in tumor progression and highlights its potential as a viable therapeutic target.

The prediction of drug sensitivity utilizing IC50 values revealed that patients belonging to the high-risk group displayed decreased responsiveness to drugs, which included Bortezomib, Olaparib, Tamoxifen, and Axitinib as opposed to the low-risk group. This suggests that patients classified as high-risk may face greater challenges in treatment and may require more personalized therapeutic approaches to overcome resistance to traditional chemotherapy agents.

What’s more, we confirmed the high expression of KRT6B in lung cancer cells through RT-qPCR. Subsequently, KRT6B was knocked down, and CCK8 assays demonstrated that KRT6B significantly enhances the proliferative capacity of lung cancer cells, thereby promoting tumorigenesis in lung cancer.

However, there are some limitations in this study. It includes the heterogeneity among different databases and the limitation of experimental conditions. In addition, because the data are from public databases, clinical information may not be comprehensive enough to fully account for individual patient differences. And these findings are only based on bioinformatics methods and lack further experimental verification. Finally, although the constructed risk prediction model performed well in multiple independent cohorts, its clinical value needs to be confirmed by further prospective studies.

In conclusion, this study not only provides a detailed analysis of heterogeneity of NSCLC cells and metabolic features but also develops a risk signature for NSCLC patients, providing robust backing for prognosis estimation and customized treatment strategies. Additionally, notable variations in gene expression patterns, immune microenvironments, and drug responsiveness were discernible among various risk groups, offering valuable insights for the identification of novel prognostic biomarkers. Addressing these limitations in future research could help confirm the results of our research.

## 5 Conclusion

To summarize, single-cell data from public databases was utilized to reveal the heterogeneity of gene expression and metabolic characteristics in NSCLC cells. Focusing on the malignant cell subpopulations, we identified four highly activated metabolic pathways. Utilizing WGCNA, we discovered modules and hub genes that have notable correlations with these metabolic pathways. Subsequently, a risk signature for NSCLC patients was constructed based on a combined TCGA dataset, and an in-depth analysis of the model followed. The NSCLC patients benefit from this model, which offers strong backing for evaluating prognosis and devising individualized treatment plans. This research not only provides a comprehensive exploration of metabolic reprogramming and its biological functions in NSCLC but also offers new insights into early diagnosis, prognostic evaluation, and targeted therapies for NSCLC, with significant clinical implications.

## Data Availability

The original contributions presented in the study are included in the article/supplementary material. Further inquiries can be directed to the corresponding authors.
